# Fluidic Force Discrimination Assays: A New Technology for Tetrodotoxin Detection

**DOI:** 10.3390/md8030565

**Published:** 2010-03-10

**Authors:** Betsy Jean Yakes, Stacey M. Etheridge, Shawn P. Mulvaney, Cy R. Tamanaha

**Affiliations:** 1 US Food and Drug Administration, Center for Food Safety and Applied Nutrition, 5100 Paint Branch Parkway, College Park, MD 20740, USA; E-Mail: stacey.etheridge@fda.hhs.gov (S.M.E.); 2 US Naval Research Laboratory, 4555 Overlook Ave, SW, Washington, DC 20375, USA; E-Mails: shawn.mulvaney.ctr@nrl.navy.mil (S.P.M.); cy.tamanaha@nrl.navy.mil (C.R.T.)

**Keywords:** tetrodotoxin, antibody inhibition assay, bioassay, Fluidic Force Discrimination, microbead labels

## Abstract

Tetrodotoxin (TTX) is a low molecular weight (~319 Da) neurotoxin found in a number of animal species, including pufferfish. Protection from toxin tainted food stuffs requires rapid, sensitive, and specific diagnostic tests. An emerging technique for the detection of both proteins and nucleic acids is Fluidic Force Discrimination (FFD) assays. This simple and rapid method typically uses a sandwich immunoassay format labeled with micrometer-diameter beads and has the novel capability of removing nonspecifically attached beads under controlled, fluidic conditions. This technique allows for near real-time, multiplexed analysis at levels of detection that exceed many of the conventional transduction methods (e.g., ELISAs). In addition, the large linear dynamic range afforded by FFD should decrease the need to perform multiple sample dilutions, a common challenge for food testing. By applying FFD assays to an inhibition immunoassay platform specific for TTX and transduction via low magnification microscopy, levels of detection of ~15 ng/mL and linear dynamic ranges of 4 to 5 orders of magnitude were achieved. The results from these studies on the first small molecule FFD assay, along with the impact to detection of seafood toxins, will be discussed in this manuscript.

## 1. Introduction

Tetrodotoxin (TTX), a low molecular weight neurotoxin, is found in a number of organisms including pufferfish, California newts, parrotfish, frogs of the genus *Atelopus*, blue-ringed octopus, starfish, angelfish, and xanthid crabs [1, 2]. This neurotoxin is believed to originate from certain bacteria including strains of *Vibrio*, *Pseudomonas* sp., and *Alteromonas* that are found associated with the host organism [3–5]. Tetrodotoxin toxicity is imparted by its high affinity to block voltage-gated sodium channels which transport Na^+^ between the exterior and interior of cells [6]. Poisoning in humans is characterized by rapid onset of numbness in the face and extremities as well as mild gastrointestinal effects, with severe poisoning culminating in respiratory paralysis and death [7]. While tetrodotoxin poisoning is relatively rare in the United States [1], in Japan where pufferfish is considered a delicacy (*i.e.*, *fugu*) there are reported to be 30–100 cases per year with a mortality of 6.4% [8].

In recent years, TTX has been found in the United States and international food stuffs. The most notable US cases include three chefs in California that became ill when they consumed pre-packaged *fugu* [9] and two people in Illinois who ate pufferfish misbranded as monkfish [10]. From these incidents, it is clear that the most common organism that impacts public health and harbors TTX is pufferfish, which accumulate TTX in the gonads, liver, intestines, and skin [1]. Correct preparation of pufferfish and consumption of the muscle alone reduces the risk of poisoning; however, the techniques for preparation are difficult and prone to error. This potential contamination of the pufferfish muscle along with the inability to inhibit TTX action by proper cooking methods (e.g., freezing for storage, heating during cooking, using acid to cook) cause TTX to be a potentially dangerous foodborne pathogen. Furthermore, the LD_50_ for TTX in mammals is 2–10 μg/kg intravenously and 10–14 μg/kg subcutaneously [11]. The only country with an established regulatory limit for TTX is Japan [12]. While there is no established action level for TTX in the US, saxitoxin (action level of 80 μg STX equivalents per 100 g tissue) is considered to be comparable, given the similar toxicities and modes of action for the two toxins.

The current standard for testing TTX in foodstuffs is the mouse bioassay [12, 13]. This test, however accurate in determining toxicity of a sample, suffers from potential ethical concerns over the use of live animals. Furthermore, the mouse bioassay does not test for a specific toxin, just the time it takes for a mouse to die following intraperitoneal injection of a sample. In this assay, the time of death is proportional to toxicity; however, the dynamic range for this proportional relationship is small. Thus, many samples must be diluted and new mice injected to yield a result that falls within the quantitative dynamic range. In addition, a sample resulting in mouse incapacitation would need further confirmatory testing to determine the exact source toxin (e.g., TTX, STX, brevetoxin, *etc*.). Therefore a method that eliminates the use of live animals, tests for the specific presence of TTX, and provides a larger dynamic range would be beneficial.

Functional methods relying on biological components (e.g., native receptors) have become popular alternatives in recent years. One such method relies on using sodium ion channels from rat brain membrane preparations and radio-labeled saxitoxin or tetrodotoxin (^3^H-STX or ^3^H-TTX) [14]. In this receptor binding assay (RBA), radio-labeled toxin and TTX compete for the receptors, with the signal then inversely proportional to sample toxicity [15, 16]. Due to the nonspecific nature of receptor binding, this assay does not have the ability to distinguish between TTX and the STX congeners. In addition, the use of radioactive components makes the method expensive, limited by radiation safety regulations, and potentially hazardous to the user and environment.

A common detection technique for foodborne toxins and small molecules is high performance liquid chromatography (HPLC) that is sometimes coupled with mass spectrometry (MS). These methods have been developed for TTX and allow for identification of the toxin and congeners that may be present [17–20]. Interestingly, recent studies have shown that both STX and TTX can be present in pufferfish, and thus using an LC/MS method can be advantageous in outbreak samples to identify the causative agent of illness [21]. However, HPLC and LC/MS can use harsh, environmentally deleterious solvents, and these techniques may not be suited to the rapid screening needed for the high number of potentially contaminated materials that would arise from a large outbreak.

Alternative assays to the RBA have been shown to be successful as rapid screening tools. These assays mainly rely on antibodies that are specific to the desired toxin and therefore allow discrimination between TTX and other commonly occurring marine toxins. Many immunoassay formats and transduction techniques have been designed with perhaps the most common being the enzyme-linked immunosorbant assay (ELISA) [22–24]. Unfortunately, ELISA can be lengthy and require a lot of labor to complete. Recently developed immunoassays that focus on flow based systems, such as surface plasmon resonance biosensors [25], show promise for more rapidly detecting TTX and work to improve these technologies is underway. Other novel detection methods for TTX include surface-enhanced Raman spectroscopy with metallic nanoparticle arrays that are able to obtain the tetrodotoxin spectrum at concentrations as low as 0.9 ng/mL [26] and electrochemical immunosensors with ng/mL detection limits following 30 min assays [27].

An emerging assay option that uses a flow-based system and antibody recognition is Fluidic Force Discrimination (FFD). In FFD assays, a sample is mixed with secondary antibodies and conjugated microbead labels in solution. Next, the analyte-loaded beads are introduced to a microarray, captured at the corresponding primary antibody, and controlled fluidic forces are applied to preferentially remove nonspecifically bound beads. The beads remaining are counted to determine the analyte identity and concentration. Using this protocol, 35 aM staphylococcal enterotoxin B (SEB) was detected in <20 min [28] with a log-linear dose response curve covering six orders of magnitude. FFD assays have also been used to detect ricin and anthrax antigens, as well as several nucleic acid targets, including genomic DNA from *Bacillus anthracis sterne*, *B. anthracis ames*, and *B. thuringiensis* [29]. Additionally, a key advantage of FFD assays is that they are compatible with complex matrices and detection has been demonstrated in biological (e.g., blood, plasma, serum, saliva, urine, feces), environmental (e.g., waste water), and food (e.g., spinach, milk, apple juice) samples.

In the work presented here, we extend FFD assays to the detection of small molecule targets. By combining a modified inhibition immunoassay with the FFD technology, a rapid and sensitive detection platform was developed. This proof of concept study not only indicated the ability of FFD to detect small molecules, specifically TTX in this case, it also showed that FFD can yield a large linear dynamic range of detection. This large dynamic range would decrease the number of times a sample would have to be diluted and run to quantitatively determine concentration thus saving time and expense. Herein, the substrate preparation, immunoassay format, detection technique, and the results obtained from preliminary studies will be discussed.

## 2. Results and Discussion

### 2.1. Immunoassay Platform and FFD Instrumentation

FFD immunoassays have been developed with a focus on using sandwich immunoassays for rapid, sensitive, and selective antigen detection. In the most basic form of these assays, an antibody is immobilized on the flow cell surface, the antigen is then captured to the surface, and the bound complex is labeled with a secondary antibody. Antibody conjugated microbeads are then added to the flow cell that recognize the secondary antibody [28]. FFD is performed and the amount of beads bound to the surface quantified. However, for small molecules such as TTX, sandwich immunoassays are not amenable to detection, as it is rarely possible to bind two antibodies to these small analytes. To alleviate the size and antibody constraints, an immunoassay platform can be designed that takes advantage of single antibody binding in an inhibition immunoassay format.

**Figure 1:**
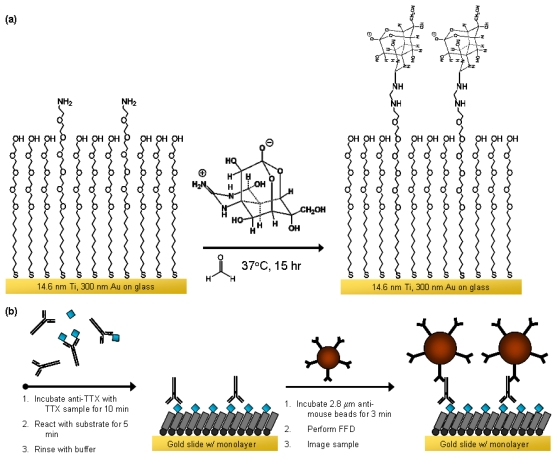
(a) Tetrodotoxin surface immobilization via formaldehyde onto an NH_2_-OEG and OH-OEG mixed thiol monolayer, and (b) FFD inhibition immunoassay where the teal diamonds represent TTX.

To design the substrate for FFD detection of TTX, modifications were made to a previously optimized biosensor platform (Figure 1a) [25]. A 1 part NH_2_-oligoethylene glycol (NH_2_-OEG) thiol is mixed with 9 parts OH-OEG thiol to create a substrate that is both bioresistant [30, 31] and amenable to conjugation of TTX [23, 32]. The immobilization reaction is performed by using the crosslinker formaldehyde to covalently attach TTX to the self-assembled monolayer (SAM). By immobilizing TTX on the surface in this manner, the CH_2_OH functionality and antibody-reactive portion of the molecule faces up and is available to bind anti-TTX. Based on this orientation then, an inhibition immunoassay can be performed where the mouse anti-TTX is captured and subsequently labeled by anti-mouse microbeads as shown in Figure 1b. Due to the nature of the inhibition immunoassay, the larger the concentration of TTX in the analyte solution, the less free antibodies there will be to bind to the substrate, and consequently the fewer microbeads observed upon readout.

**Figure 2:**
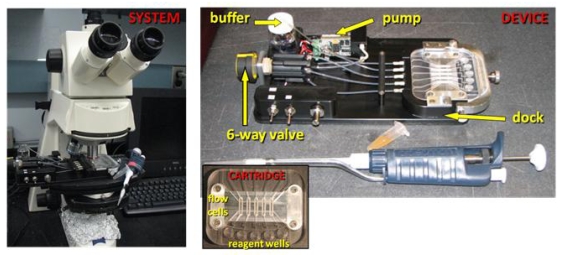
FFD instrument mounted on an upright microscope (System image). The functionalized substrate is held in the compression fit cartridge, and reagents are added and removed via the reagent wells in front of each of five individually addressable flow cells (Cartridge image). A peristaltic pump and six-way valve determine which flow cell is operational (Device image).

FFD assay instrumentation is shown in Figure 2. All assay steps occur in a microfluidic cell formed when the functionalized surface is mounted in a compression fit cartridge. This custom built platform mounts on an upright microscope and has five individually addressable sensing areas per substrate. Using a peristaltic pump in either push or pull mode, buffer and reagents can be added to and removed from the sensing areas. Following FFD, captured beads are imaged with low power microscopy (5×), and images are captured on a CCD camera. The bench top instrument shown here is a manual workhorse, well designed for assay development; however, in the future it should be feasible to develop an automated, shoe-box sized, portable instrument. Most of the components of this system have already been developed for the compact Bead Array Sensor System (*cBASS*^®^), a field portable bioassay platform employing FFD assays and using magnetoresistive sensors to detect captured magnetic bead labels [33].

### 2.2. TTX Immunoassay Results

**Figure 3:**
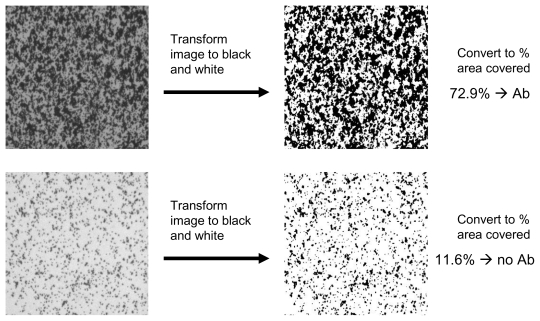
Far left: Images of surface after FFD performed for positive control (Ab, top image) and negative control (no Ab, bottom image). Images are then transformed using Image J software.

After performing the inhibition immunoassay with TTX, the substrate is imaged through a 5× microscope objective, and the image is captured with a CCD camera. To interrogate the quantity of microbeads that are immobilized on the surface, the image is processed using Image J software (Figure 3). The images on the far left represent raw data files where the top image has high coverage (antibody present), and the bottom image has low coverage (no antibody). The images are transformed with the software to yield a pure black and white representation of the surface. By using the software, the percent area covered by dark space is then determined, and this value is representative of microbead binding. From these data, it is seen that at full coverage, the substrate had approximately 73% of the surface covered by microbeads. At the other extreme, when there was no antibody (Ab) present in the assay, the substrate exhibited about 12% coverage. This allows a range of 61% for the immunoassay calibration curve. To determine the linear dynamic range and assay sensitivity, the assay is then performed by mixing various concentrations of TTX with the antibody in the immunoassay format shown in Figure 1b, and the data processing is performed as described herein.

**Figure 4:**
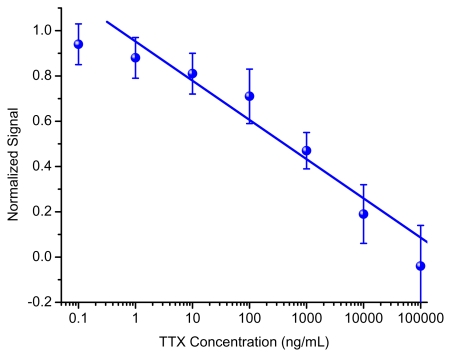
Standard curve for TTX immunoassay with the normalized response versus TTX concentration used in the immunoassay. The number of replicate measurements for each concentration (N) is 2 (0.1, 100,000 ng/mL), 4 (1000 ng/mL), 5 (10,000 ng/mL), and 6 (1, 10, 100 ng/mL). Curve has 4 to 5 orders of linear dynamic range (between 1 and 100,000 ng/mL) with a fit of R^2^ = 0.969, y = −0.1732 log(x) + 0.9523. Variation in N is due to substrate availability and the desire to perform more replicates near the limit of detection and in the linear dynamic range.

Upon completion of these immunoassays and normalization of the data, the results can be shown in a calibration curve (Figure 4). As expected, when TTX concentration is low there are many antibodies available to bind to the surface, resulting in a larger number of beads and thus a high signal. The inverse is true in which a high TTX concentration consumes the antibodies in solution, and therefore no signal is seen. The curve, ranging from 1 to 100,000 ng/mL TTX, shows a linear trend when the normalized signal is plotted with respect to the log of TTX concentration. This large dynamic range may offer advantages over other techniques as fewer sample dilutions would be necessary to determine an unknown concentration. Furthermore, the sensitivity of the assay, as defined by the IC_20_ (inhibitory concentration of 20% at 0.8 normalized signal), is 14 ng/mL which compares well to HPLC and LC/MS methods.

One drawback seen in the current data arises from the high standard deviations of each concentration. These relative errors, on the order of 10–15%, could lead to a less quantitative assay. We attribute these errors to nonspecific adsorption of the microparticles to the gold surface, as previous FFD experiments using non-gold surfaces yielded much lower standard deviations [28, 33]. The gold surface, however, is not essential for FFD performance and was only utilized because of the well-established surface chemistry for TTX on gold platforms [25]. Current research is focused on redesigning the substrate conjugation chemistry to eliminate the gold platform and decrease the error while maintaining the specificity, sensitivity and large dynamic range of this assay.

Further experiments indicated an advantage to the TTX immunoassay not previously incorporated into FFD assays. In surface plasmon resonance biosensors, assay platforms can be reconditioned by removing the antibody from the substrate at the end of each experiment [34] which can minimize error, reduce cost per sample, and decrease analysis time. This potential regeneration of substrates was investigated by immersing the used surfaces in a 50 mM NaOH solution and placing on a shaker for 1 hr. After rinsing the slides with water and drying with N_2(g)_, it is expected that the microbeads and anti-TTX will be removed from the surface leaving a substrate with just TTX immobilized to the SAM. Upon regeneration and running further immunoassays on an individual slide, only minor changes in nonspecific binding (5%) and signals (7.5%) that appeared uniform throughout additional regeneration cycles were observed. This process then also appears to add a source of error in that regeneration was not complete. As such, regeneration was found to be feasible but the optimal conditions, such as using acidic regeneration solutions to avoid potential base sensitivity of TTX, are being investigated as an area of improvement for this immunoassay.

## 3. Experimental Section

### 3.1. Materials

Anti-TTX monoclonal antibody (1 mg lyophilized protein) was purchased from Hawaii Biotech, Inc. (Aiea, HI), and TTX was procured from Sankyo Co, Ltd (Tokyo, Japan). Monobasic potassium phosphate (100mM), dibasic potassium phosphate (100 mM), 37% formaldehyde solution, triethylamine, and glacial acetic acid were obtained from Sigma-Aldrich (St. Louis, MO). HS-(CH_2_)_11_-EG_4_-OH and HS-(CH_2_)_11_-EG_6_-NH_2_ hydrochloride were synthesized by ProChimia Surfaces (Sopot, Poland). Ethanol (absolute, anhydrous, ACS/USP grade) was purchased from Pharmaco AAPER (Shelbyville, KY). 50% w/w sodium hydroxide solution was procured from J. T. Baker (Phillipsburg, NJ). Sheep anti-mouse, 2.8 μm Dynabeads were purchased from Invitrogen (Carlsbad, CA). Gold substrates (146 Å titanium adhesion layer, 3000 Å gold on glass microscope slides) were prepared using the AJA Sputtering Unit at the University of Maryland Nanocenter (College Park, MD). All water used in these experiments was 18.2 MΩ·cm (Millipore MilliQ Academic, Billerica, MA).

### 3.2. Substrate Preparation

A mixed SAM of oligoethylene glycol (OEG) alkanethiol (hydroxyl-terminated and amine-terminated forms) on gold is prepared by modifying a procedure to form TTX surfaces for surface plasmon resonance biosensors [25]. This monolayer allows for covalent linking of tetrodotoxin to the surface (NH_2_-OEG) via formaldehyde coupling [23, 32] as well as minimization of nonspecific protein adsorption (OH-OEG) [30, 31]. Gold slides are ozone cleaned (20 min, NovaScan PSD-UV, Ames, IA), rinsed with water and then ethanol, and dried with N_2(g)_. The dry slides are immersed into the mixtures (described next) for 18 hours. Briefly, 5 mM ethanolic solutions of each thiol are prepared. To generate the SAM with a 1:9 mixture of NH_2_-OEG thiol to OH-OEG thiol, 46.5 mL ethanol, 1.5 mL triethylamine, 0.2 mL NH_2_-OEG thiol, and 1.8 mL OH-OEG thiol are mixed together. The triethylamine addition to the SAM formation allows for a smoother surface with less unbound thiols [35].

After monolayer formation, the slide is removed from solution, rinsed with ethanol, 10% acetic acid in ethanol, followed by ethanol and dried with N_2(g)_. This substrate is then reacted with a mixture of 932.6 μL 100 mM pH 7.0 phosphate buffer, 41.8 μL of 10 mg/mL TTX, and 69.6 μL 37% formaldehyde. The formaldehyde functions as a one-carbon crosslinker which is able to covalently bind the TTX to the thiolate surface. Furthermore, this conjugation does not tie up the antibody binding motif located on the CH_2_OH side of TTX [25]. The reaction proceeds for 72 hrs at 37 °C, and after formation, the substrates may be stored at 4 °C until use. Upon removal, the chip is rinsed with DI water and dried with N_2(g)_.

### 3.3. FFD Setup

FFD assays were performed with a custom built instrument mounted on an upright microscope. The TTX-functionalized substrate is housed in a compression-sealed fluid cartridge with five individually addressable flow cells [36]. At the front of each flow cell is a 200 μL well used for introduction and exchange of reagents. The opposing side of the cartridge is connected to a buffer reservoir and peristaltic pump. FFD substrate conditioning begins by pumping buffer through the cartridge to the base of the reagent well.

### 3.4. Inhibition Immunoassay

As TTX is a small molecule that is challenging to detect directly, an inhibition immunoassay was employed. One part TTX sample in buffer (1× PBS, 5% Carnation dried skim milk) is mixed with 9 parts 1.1 μg/mL anti-TTX in buffer for 10 min. This inhibition assay mixture is then pulled into the FFD cartridge until the flow cell is filled, and the mixture is exposed to the TTX-functionalized surface for 5 min. During this time, antibodies not occupied with TTX antigen from the sample can bind to the TTX molecules on the substrate. Next, the mixture is pumped out of the cartridge and removed from the reagent well which simultaneously refills the flow cell with buffer. Then, the 2.8 μm diameter beads conjugated with sheep anti-mouse antibodies are pulled into the flow cell and allowed to settle for 3 min. This allows the beads to bind to any surface-bound TTX monoclonal antibodies. Finally, buffer is pumped through the flow cell at a controlled speed to remove nonspecifically attached beads, as previously described in detail [28, 33]. For each substrate, which had five sample flow cells, one cell is used as a positive control (no TTX, Ab), one cell as a blank (no TTX, no Ab), and three cells as immunoassay samples (TTX and Ab).

### 3.5. Data Processing

Beads remaining after the FFD assay are imaged with low power (5×) optical microscopy, and images are captured with a CCD camera. Using Image J software (http://rsbweb.nih.gov/ij/index.html), the images are flattened, thresholded, and digitized to distinguish beads and background. A fixed area (100 × 100 pixels) is selected, and the percent area of beads determined. For each channel, an average of five images was taken, and the coverage of beads determined. The images are then compared to both a blank signal (*i.e.*, no TTX and no Ab) and a no inhibition, positive control signal (*i.e.*, no TTX but Ab in solution) on the same assay substrate. For uniformity between chips, the average sample signal had the nonspecific adsorption, as indicated in the blank run average signal, subtracted. Next, this result was divided by the positive control average signal that is due to just antibody binding. This process yields a normalized signal response. These normalized values for a single concentration with multiple sample runs (as indicated by N) are averaged together and plotted versus original concentration of TTX in the sample prior to antibody dilution.

## 4. Conclusions

This work has demonstrated the first competitive FFD immunoassay. The FFD immunoassay has the potential to detect TTX in complex matrices by using a sensitive, selective assay surface and controlled fluidic detachment of nonspecifically bound microparticles. In these studies, a substrate with covalently linked TTX was used as a platform for inhibition immunoassays that allowed for 4–5 orders of linear magnitude and detection limits of approximately 15 ng/mL. This rapid, sensitive platform could decrease sample preparation and run times thus allowing for a superior screening assay to current methods. Research is underway to improve the current immunoassay by decreasing the standard deviation in replicates through better surface formation and regeneration.
